# LptD is a promising vaccine antigen and potential immunotherapeutic target for protection against *Vibrio* species infection

**DOI:** 10.1038/srep38577

**Published:** 2016-12-06

**Authors:** Zhenzhong Zha, Chuchu Li, Weiyan Li, Zhicang Ye, Jianyi Pan

**Affiliations:** 1 School of Life Sciences, Zhejiang Sci-Tech University, Hangzhou 310018, China; 2 Zhejiang Provincial Key Laboratory of Silkworm Bioreactor and Biomedicine, Hangzhou 310018, China

## Abstract

Outer membrane proteins (OMPs) are unique to Gram-negative bacteria. Several features, including surface exposure, conservation among strains and ability to induce immune responses, make OMPs attractive targets for using as vaccine antigens and immunotherapeutics. LptD is an essential OMP that mediates the final transport of lipopolysaccharide (LPS) to outer leaflet. The protein in *Vibrio parahaemolyticus* was identified to have immunogenicity in our previous report. In this study, broad distribution, high conservation and similar surface-epitopes of LptD were found among the major *Vibrio* species. LptD was further revealed to be associated with immune responses, and it has a strong ability to stimulate antibody response. More importantly, it conferred 100% immune protection against lethal challenge by *V. parahaemolyticus* in mice when the mice were vaccinated with LptD, and this finding was consistent with the observation of efficient clearance of bacteria in vaccination mice. Strikingly, targeting of bacteria by the LptD antibody caused significant decreases in both the growth and LPS level and an increase in susceptibility to hydrophobic antibiotics. These findings were consistent with those previously obtained in *lptD*-deletion bacteria. These data demonstrated LptD is a promising vaccine antigens and a potential target for antibody-based therapy to protect against *Vibrio* infections.

*Vibrio* spp. are Gram-negative bacteria that commonly reside in estuaries and coastal waters worldwide. Although most of these bacteria are harmless, some species can infect a broad spectrum of fish and mammals and therefore cause serious disease. Among these pathogenic species, *V. parahaemolyticus*, *V. harveyi* and *V. alginolyticus* have been considered to be the major causative organisms of diseases in marine fish and shellfish^1^,^2^, leading not only to serious economic loss but also to global increase in foodborne illnesses derived from the consumption of raw or undercooked seafood^3^. Currently, antibiotics remain the major measures to control these bacterial infections, both in aquaculture and in clinical treatment. However, the widespread and frequent use of antibiotics has led to the rapid development and spread of antibiotic resistance in *Vibrio* species^4^,^5^,^6^,^7^,^8^, which has become a serious threat to public health worldwide. Therefore, it is urgent to develop prevention strategies or alternative treatments to protect against bacterial infections. Although effective vaccination has been suggested to be the most efficient and economical method to prevent and control infection^1^,^2^, few commercial vaccines are available currently^9^. In addition, antibody-based therapies, which have caused a therapeutic revolution in the fields of oncology and rheumatology, have been suggested to provide new, effective therapies, but they remain severely underdeveloped for the treatment of infectious diseases^10^,^11^.

Outer membrane proteins (OMPs) of Gram-negative bacteria have been suggested to be good targets for vaccine and drug development against bacterial infections^12^,^13^,^14^,^15^. Several OMPs of *V. parahaemolyticus*, including OmpW, OmpV, OmpU, OmpK^16^, PsuA, PvuA^17^, VPA1435, VP0764, VPA1186, VP1061 and VP2850^18^, have been reported to provide immune protection against the bacteria infection in laboratory trials. Moreover, our previous study identified several OMPs, LptD, LamB, OmpA, OmpK, OmpU, VP0802, VP1243 and VP0966, to be immunogenic proteins from *V. parahaemolyticus* using an immunoproteomics approach^19^. Among these proteins, LptD appeared to be a highly immunogenic protein. The structure and function of LptD in some Gram-negative species have been studied intensively in recent years. LptD is an integral OMP, and its crystal structure, which forms a 26-strand β-barrel, the largest β-barrel reported so far, was revealed by Dong *et al*.^20^ and Qiao *et al*.^21^ at almost the same time two years ago. LptD and other 6 proteins – LptA, B, C, E, F and G – constitute a trans-membrane complex responsible for transporting lipopolysaccharide (LPS)^22^,^23^,^24^. Through interaction with the lipoprotein component LptE, which adopts a roll-like structure embedded in the LptD barrel, LptD mediates the final translocation of fully synthesized LPS from periplasm to the outer leaflet of the outer membrane^25^,^26^,^27^,^28^,^29^,^30^. LPS, a glycolipid consisting of several fatty acyl chains and perhaps hundreds of sugars, is synthesized in the cytoplasm and finally assembled in inner membrane. As a major component of the outer membrane of Gram-negative bacteria, LPS not only plays critical roles in protecting organisms from harsh environments and in colonizing the host and evading attacks from the host immune system, but it also forms a permeation barrier, which prevents hydrophobic antibiotics from entering the organism, thus conferring antibiotic resistance^25^,^27^,^28^. Therefore, LptD could be an attractive target for the development of novel vaccines or therapeutics.

In the current study, we showed that LptD was highly conserved and shared similar surface epitopes among *Vibrio* species. The protein has a strong ability to introduce antibodies in animals, and it confers extremely efficient immune protection against *Vibrio* infection. Notably, by targeting the LptD antibody, bacteria showed a significant reduction in growth and LPS level and increased susceptibility to antibiotics. The results suggested for the first time that LptD is a promising target for the development of effective vaccines and antibody-based therapies to control *Vibrio* infection.

## Results

### Increasing expression of LptD during bacteria incubation with fresh serum

**Figure 1:**
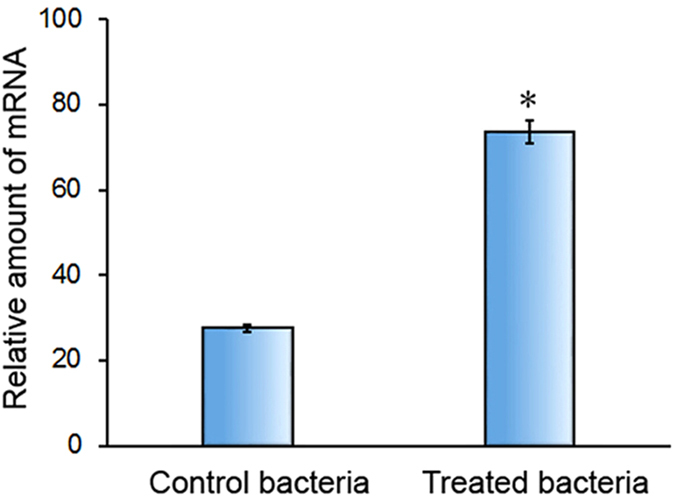
Increasing expression of LptD during bacteria exposure to serum. *V. parahaemolyticus* cells were treated by incubation with 15% fresh human serum for 1 h at 37 °C. The relative mRNA transcriptional levels of LptD were quantified by real-time quantitative PCR using primers of F (CCCGAACATTCCAGACGAGACAA) and R (CGTACTGAACGCCACCGTGATAGA), and 16 S RNA was analyzed simultaneously as an internal control. The data were acquired from three independent assays. Statistical significance (*P* < 0.01, n = 3) is indicated by an asterisk.

The key steps in the development of vaccines could be the identification of antigens. OMPs have been shown represent good candidates for scanning vaccine antigens^19^,^31^,^32^, likely due to their presentation to the host to elicit an immune response during the infection^19^,^33^,^34^. In our previous work, LptD was identified as an immunogenic OMP by immunoproteomic techniques^19^. To explore further the possibility of using LptD as a potential vaccine candidate or antibody therapeutic target, we first determined the variations in LptD in bacterial responses to serum by real-time quantitative PCR. The results showed that, after bacteria incubation with fresh serum for 1 h, the relative expression level of LptD was 73.53 ± 2.67, while the value was as low as 27.50 ± 0.80 for control bacteria that were not incubated with fresh serum (Fig. 1). The significantly increased expression of LptD during bacterial exposure to fresh serum suggested that the protein might play an important physiological role in bacterial response to hosts, although the detailed mechanism in the process requires further analysis, indicating that LptD could be a potential target.

### The conservation, distribution, structure and epitopes analysis of LptD

**Figure 2:**
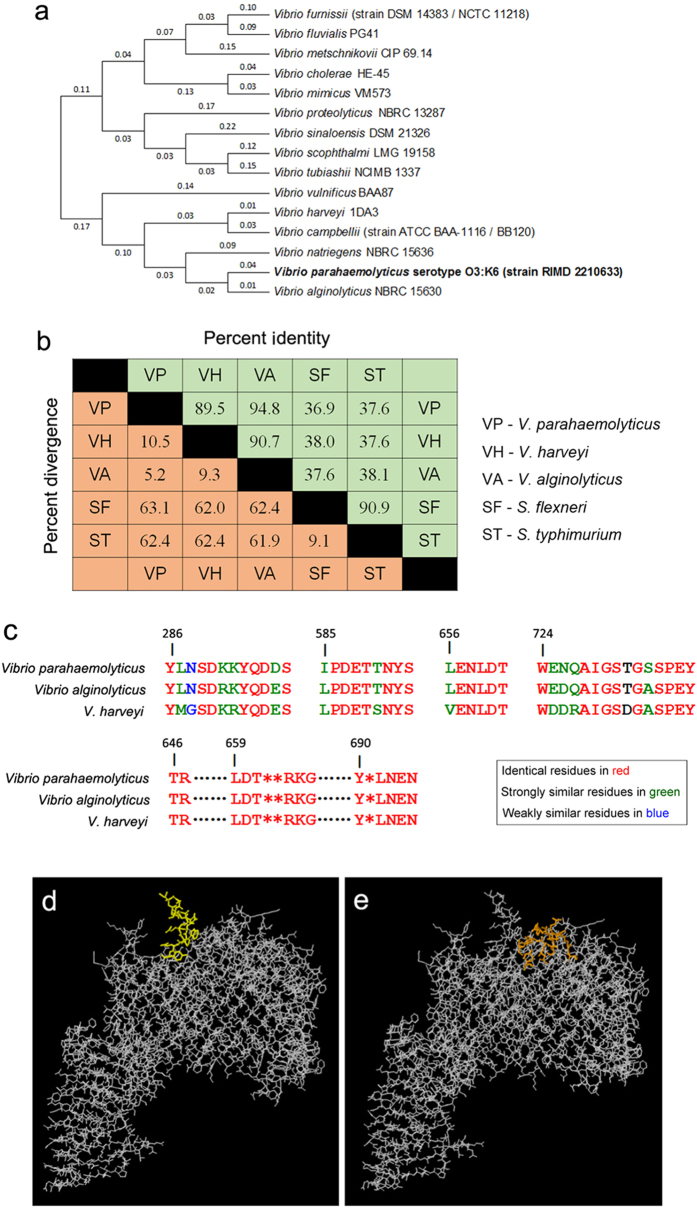
Bioinformatics analysis of LptD based on the amino acid sequences. (**a**) A phylogenetic tree based on Lptd sequences was constructed using MEGA software, version 6.0, based on the maximum likelihood method. (**b**) Percentage similarities and divergences of the LptD sequences from other Gram-negative bacteria. The sequence pair distances of amino acid residues were determined by ClustalW. (**c**) Homology alignment of four linear antibody epitopes – YLNSDKKYQDDS (286–297) (score of 0.77), IPDETTNYS (585–593) (score of 0.648), LENLDT (656–661) (score of 0.507) and WENQAIGSTGSSPEY (724–738) (score of 0.78) – and the discontinue epitope T646R647…L659D660T661**R664K665G666…Y690*L692N693E694N695 among major Vibrio species. Epitopes of *V. parahaemolyticus* LptD were predicted by the ElliPro online server, based upon the modelled three-dimensional structure of LptD. Identical residues are in red, strongly similar residues are in green, and weakly similar residues are in blue. The three-dimensional structure shows (**d**) a representative, predicted linear epitope of WENQAIGSTGSSPEY (724–738) (epitope residues are in yellow) and (**e**) a discontinuous epitope of T646R647…L659D660T661**R664K665G666…Y690*L692N693E694N695 (epitope residues are in orange).

**Figure 3:**
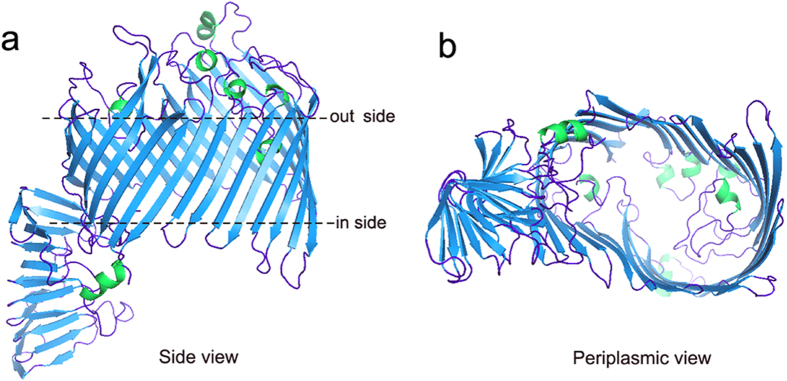
The modelled three dimensional structure of LptD. The three-dimensional structure was modelled using automated homology prediction with the Phyre V2.0 online server, based on the template c4q35A, and 741 residues (11–752, 97% of sequence coverage) were modeled with 100.0% confidence. The structure figure was rendered using PYMOL software. (**a**) Side view of the structure. (**b**) View of the structure from the periplasmic side.

To explore the possibility of using LptD as a potential versatile vaccine candidate, the signal peptide-truncated amino acid sequence of *V. parahaemolyticus* LptD was submitted for bioinformatics analysis. The sequence conservation of LptD was analyzed both among *Vibrio* species and across other typical Gram-negative bacteria. The results showed that LptD was widely distributed and conserved in all 138 *Vibrio* species with sequence identity from 55 to 100% (data not shown). The sequence identities were especially close to those of *V. alginolyticus* (94.8%) and *V. harveyi* (89.5%), as well as the major pathogenic *Vibrio* species. A phylogenetic tree based on the LptD sequence with high sequence identities is shown in Fig. 2a. In addition, the sequence conservation of *V. parahaemolyticus* LptD across other Gram-negative bacteria was also assayed, and relatively high homology was also found. There was 36.9% and 37.6% identity with that of *S. flexneri* and *S. typhimurium*, respectively (Fig. 2b). Three-dimensional crystal structures of LptD in these two species have both been recently determined^20^,^21^. To understand the structural features of LptD in *V. parahaemolyticus*, the amino acid sequence of the protein was subjected to structural modeling using automated homology prediction. The three-dimensional structure was predicted to form a 26-stranded β-barrel with several loops and helixes exposed to the external surface (Figs 3 and 2d and e), very similar to the structures determined in *S. typhimurium* and *S. flexneri*.

Moreover, the antibody epitopes were predicted by the ElliPro online server, based upon the modelled three-dimensional structure of LptD. There were several potential linear epitopes, such as YLNSDKKYQDDS (286–297), IPDETTNYS (585–593), LENLDT (656–661) and WENQAIGSTGSSPEY (724–738), and discontinue epitopes, such as T646R647…L659D660T661**R664K665G666…Y690*L692N693E694N695, that were predicted with relatively high scores. These potential antibody epitopes were all located on the outer surface of the modelled structure (Fig. 2d,e and Supplementary Fig. S1). When B cell epitopes were predicted by DiscoTope, similar results were observed (Supplementary Fig. S2). Notably, amino acid residues of these potential epitopes were highly conserved among the three major *Vibrio* species (Fig. 2c). These results, taken together, indicated that LptD, might possess the ability to raise antibodies with cross-immune reactions in hosts. In addition, cytotoxic T cell (CTL) epitopes were also predicted. The top 5 scoring peptides – IKYGRPFYL, IMDVPVFYV, KEENRPYRL, TLHQQDNVV and EYIEDTIIL – were predicted to be epitopes. Homologous peptides were also found in other *Vibrio* species (Supplementary Fig. S3). In conclusion, the sequence and structural features of LptD allowed it to be a potentially versatile vaccine candidate.

### Clone, expression and purification of LptD

**Figure 4:**
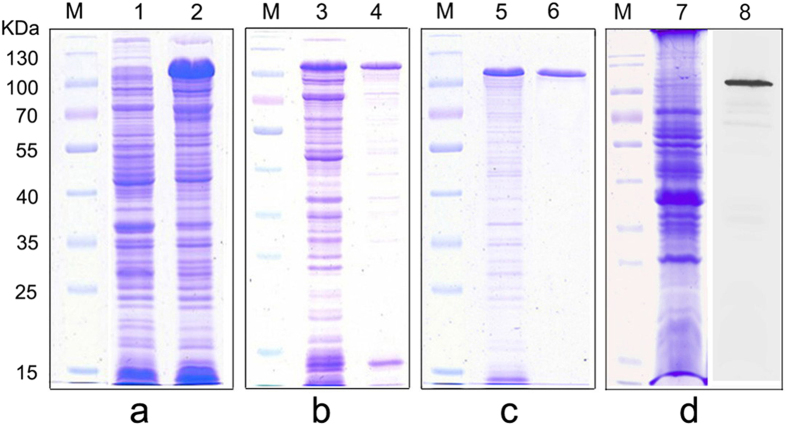
Expression and purification of recombinant LptD and determination of antibody specificity by SDS-PAGE and western blotting. (**a**) Whole cell lysates of *E. coli* BL21 (DE3) harboring recombinant plasmid of pET28a-*lptD*, either with (lane 2) or without (lane 1) IPTG induction. (**b**) The supernatants (lane 3) and pellets (lane 4) of the plasmid pET28a-*lptD* harboring-bacteria with IPTG induction after ultrasonic lysis of cells. (**c**) Purification of recombinant LptD (lane 6) from the pellets (lane 5) of (b) by Ni^2+^-NTA affinity chromatography under urea-denaturing conditions. (**d**) SDS-PAGE (lane 7) and western blotting (lane 8) analysis of membrane proteins extracted from *V. parahaemolyticus*, and antiserum from mouse #4 was used as the primary antibody. Lane M, molecular weight marker.

To investigate further the immunological characteristics of LptD, we cloned, expressed and purified the recombinant LptD protein. The *lptD* was cloned and transformed finally into host cell *E. coli* BL21 (DE3). The whole cell lysates of the resulting bacteria were analyzed by SDS-PAGE. A prominent protein band, corresponding to the position of recombinant LptD, was present in the inducing bacteria, while the corresponding band was not observed in the non-inducing bacteria, as shown in Fig. 4a, demonstrating that LptD was successfully cloned and expressed. In addition, according to the SDS-PAGE of supernatants and pellets of induced bacteria, the expressed recombinant LptD was present both in non-dissolving inclusion bodies and in dissolving solutions (Fig. 4b). Given the high percentage of recombinant LptD in inclusion bodies, the recombinant LptD were then purified from inclusion bodies. The purified protein was characterized as a single protein band by SDS-PAGE (Fig. 4c).

### Stimulation of antibody responses and protective efficacy against *V. parahaemolyticus* challenge in mice

**Figure 5:**
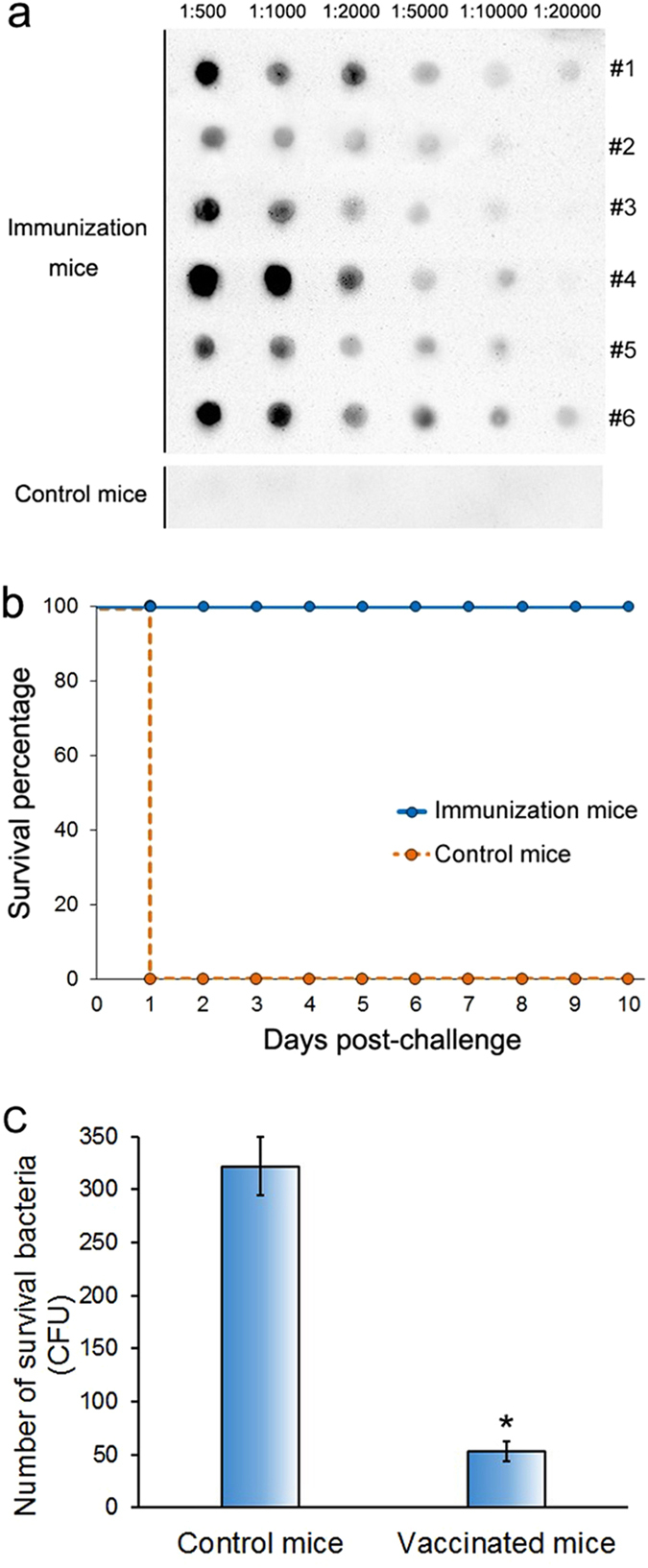
Protective immune response of LptD against lethal challenges of *V. parahaemolyticus* in mice. (**a**) Determination of the antiserum titer of each mouse in the vaccinated group (immunized with purified recombinant LptD emulsification with Freund’s adjuvant) and the control group (immunized with PBS alone and emulsification with Freund’s adjuvant) by dot-ELISA assay. The serum titers of all 6 mice in the vaccination group and a representative titer of the control group of mice are shown. (**b**) The survival percentage of the two groups of mice were evaluated following i.p. injection of 2.5 × 10^7^ CFU of *V. parahaemolyticus*. Mouse survival was observed daily for 10 days after bacterial challenge. (**c**) Bacterial clearance analysis of mice vaccinated with LptD at 1 h post-challenge with *V. parahaemolyticus*. The data were acquired from three independent assays. Statistical significance (*P* < 0.01, n = 3) is indicated by an asterisk.

Active immune protection assay was used to determine the immunoprotective efficacy provided by LptD. Therefore, the purified recombinant LptD was emulsified and immunized into mice to allow the mice to raise antibodies. The antibody specificity was measured by SDS-PAGE and western blotting analysis, which showed a single apparent band that was consistent with the LptD position (Fig. 4d). The titers of antibodies against LptD were assayed by dot-ELISA. As shown in Fig. 5a, the results showed that the antibody titers of all 6 immunized mice were at least of 1:5000, while, as expected, no apparent black dot was observed when the serum dilution ratio ranged from 1:500 to 1:20000 for control mice immunized with PBS emulsified alone, indicating that no specific antibody against LptD was present in normal serum. Together, these results verified that LptD is an immunogenic protein, and it could induce strong immune responses.

After determination of antiserum titers, all of the mice of both in immunization group and control group were lethally challenged with *V. parahaemolyticus* to observe their mortality. All of the control group mice died on the first day (to be exact, within 5 h) post-challenge, whereas all of the mice in the immunization group survived for more than the 10-day observation period (Fig. 5b). This 100% protection against infection demonstrated clearly that LptD possesses extremely high ability to stimulate protective immune responses. In addition, the bacteria-clearance efficiency of the two groups of mice was further tested, and it showed that the vaccinated mice had remarkably more efficient clearance of bacteria than the control mice (Fig. 5c). The results were consistent with the alteration of survival rates between the two groups of mice.

### Reducing the bacterial growth rate and LPS level in the presence of antibodies against LptD

**Figure 6:**
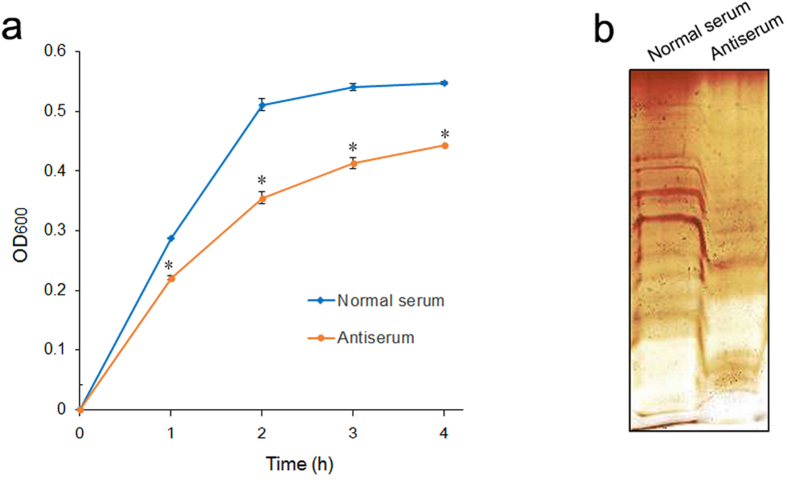
Treatment of antiserum reduces the bacterial growth and LPS level. (**a**) The growth of *V. parahaemolyticus*. A single colony of the bacterium was inoculated in MLB medium containing heat-inactivated normal mouse serum and mouse antiserum (titer of antibody against LptD was 1:10000) and was grown at 37 °C with shaking at 180 rpm. OD_600_ values at different time points were determined by spectrophotometry. Statistical significance at each time point is indicated by an asterisk (*P* < 0.01, n = 3). (**b**) Silver-stained LPS profiles of *V. parahaemolyticus*. A single-colony of inoculum cultured in MLB containing heat-inactivated normal mouse serum and mouse antiserum (antibody titer against LptD was 1:10000), respectively. Equal amounts of bacterial cells (2 × 10^9^ CFU) were used to extract LPS using a hot aqueous-phenol method, and the same volume of obtained LPS samples was submitted to electrophoresis on a slab of polyacrylamide gel and was visualized by silver staining.

There have been several previous studies finding that the deletion of *lptD* impacted the growth rate and reduced LPS levels in Gram-negative bacteria^26^,^35^,^36^. However, whether deficiency in LptD by targeting specific LptD antibodies on the bacterial surface would have similar impacts on bacteria remains unknown. Therefore, to evaluate whether antibody targeting of LptD would reduce bacterial growth and LPS levels, a single-colony inoculum was cultured in medium containing antiserum collected from vaccinated mice and normal serum (used as a control), respectively. To measure the bacterial growth, the OD values at 600 nm were detected at intervals. The results showed that bacteria in the presence of LptD antibodies grew more slowly than bacteria in the absence of the antibody (Fig. 6a). Moreover, to measure the impact on LPS levels, LPS samples were extracted from bacterial cells during growth to exponential phase and were visualized by gel electrophoresis and silver staining. As expected, the LPS level of bacteria cultured in the presence of the antibody was significantly lower than that of the bacteria grown in the absence of the antibody (Fig. 6b). These results indicated that the specific antibody targeting LptD had a similar impact on bacteria when *lptD* was deleted, suggesting that LptD could be a promising target for antibody-based therapies.

### Antibiotic susceptibility to three antibiotics during *V. parahaemolyticus* treatment with LptD antibody

**Figure 7:**
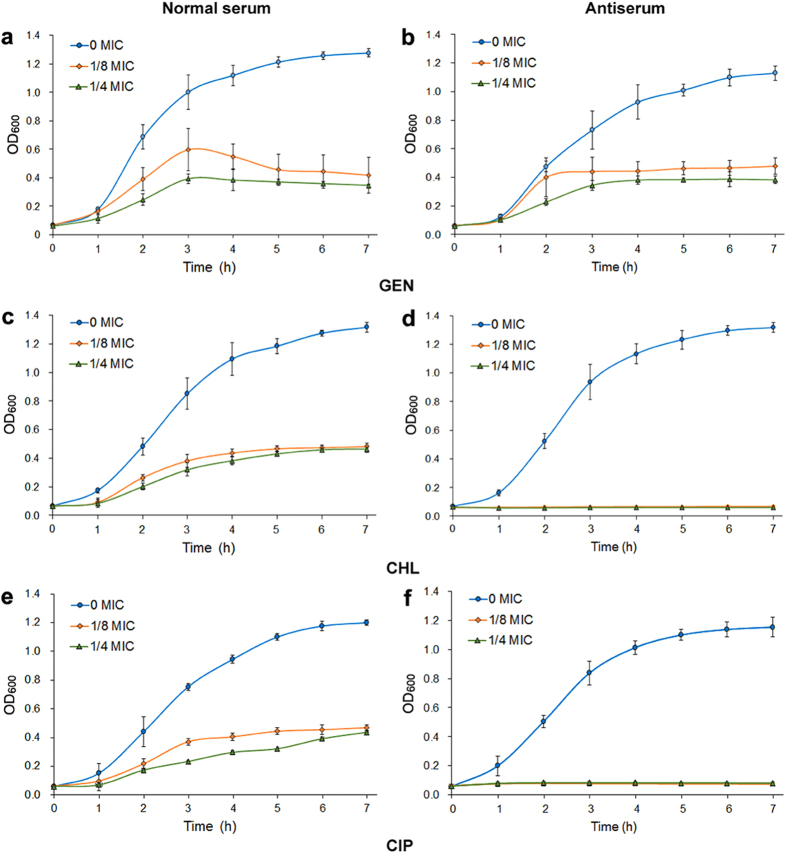
Antibiotic susceptibility of *V. parahaemolyticus* during treatment with antiserum in the presence of three antibiotics. Bacterial cells grown to exponential phase (OD_600_ of 0.5) were harvested and incubated separately with normal mouse serum and antiserum (antibody titer against LptD was 1:10000) for 1 h at 37 °C. Treated cells were recollected and adjusted to an OD_600_ of 0.3 with MLB medium. One hundred microliters of the bacterial suspensions were added to each well that contained the same volume of MLB medium supplemented with 5 μL of serum and with 1/8, 1/4, and 0 MIC (means without antibiotic) of GEN, CHL and CIP, respectively. The plates were maintained at 37 °C and the OD_600_ values were determined every 1 h. (**a**,**c**,**e**) Medium contained 5 μL of normal serum was added into each well; (**b**,**d**,**f**) Medium contained 5 μL of antiserum against LptD was added into each well.

Various studies have shown that the lack of LPS could increase outer membrane permeability and susceptibility to hydrophobic antibiotics^26^,^37^,^38^ because LPS provide a barrier against hydrophobic antibiotics^38^. Owing to the dramatically decreased LPS in bacteria by targeting of the LptD antibody that was observed, we next assayed the impact of antibiotic susceptibility. To achieve this aim, we first determined the MICs of three antibiotics – a hydrophilic antibiotic, gentamicin sulfate (GEN), and two hydrophobic antibiotics, chloramphenicol (CHL) and ciprofloxacin (CIP) – against *V. parahaemolyticus*. The MIC values for GEN, CHL and CIP were examined to be 16.0, 6.25 and 8.0 μg/mL, respectively. To evaluate the variations in susceptibility to these antibiotics, the survival capability of bacterial growth in combating by specific antibody, instead of MIC, was determined as described previously with minor modifications because measurement of survival capability is more sensitive than determination of MIC^39^. Therefore, bacterial cells were separately treated with normal serum and antiserum and then were cultured in medium containing serum and supplemented with or without antibiotics (each at a concentration of 1/8, 1/4 and 0 MIC, respectively). The bacterial survival capabilities were examined by monitoring the OD600 values of viable cells at intervals, and the results are shown in Fig. 7. We found that all bacteria were viable and could grow when they were treated with normal serum and incubated in the presence of both normal serum and different concentrations of each antibiotic (Fig. 7a,c and e). In contrast, when bacteria were treated with antiserum and grown in medium containing both antiserum and 1/8 and 1/4 MICs of CHL or CIP, bacterial survival was not observed over the 7-h experimental period (Fig. 7d and f), indicating that the bacteria were entirely inhibited, and these significant changes in bacterial survival capability suggested increasing susceptibility to CHL and CIP in bacteria targeted by the LptD antibody. However, when GEN was used for this assay, the bacteria survived for at least a 7-h period and showed slightly similar growth at both 1/8 and 1/4 MIC of the antibiotic (Fig. 7b). These results were in agreement with previous findings obtained in *lptD* deletion mutants^26^,^37^,^38^.

## Discussion

The increasing emergence of resistance to antibiotics worldwide has caused serious threats to both human health and animal breeding. Therefore, the development of efficient vaccines and alternative therapies to substitute for antibiotic therapies has been a significant concern around the world. In recent years, genetically conserved OMPs have attracted extensive attention for their potential vaccine candidates. The features of OMPs that external sequences expose to the extracellular environment not only instill these proteins with essential physiological and/or pathological functions but also might act as epitopes to induce specific immune responses^12^. For these reasons, there has been growing interest in the development of vaccines focusing on OMPs.

In our previous study, LptD, as well as other three OMPs – VP0802, VP1243 and VP0966 – with relatively low abundance, were identified as immunogenic proteins for the first time using immunoproteomics from *V. parahaemolyticus*^19^. These newly identified immunogenic proteins, along with other immunogenic OMPs identified previously, such as OmpU, OmpK, OmpA(I), OmpA(II) and LamB^18^,^40^,^41^,^42^, are palpably potential targets for the development of vaccines or novel therapeutics. In this study, we provided the first evidence that LptD might be one of the most promising vaccine candidate and/or an ideal therapeutic target for protection against *Vibrio* infection, due to it not only conferring highly efficient protective immune responses but also dramatically decreasing bacterial growth and LPS levels and increasing susceptibility to hydrophobic antibiotics during bacterial treatment with antibodies against LptD.

The OMP LptD, formerly known as OstA or Imp, has been subjected to in-depth investigation in *E. coli* (see reviews of refs 28 and 43), and it has also been subjected to studies in several other Gram-negative bacteria, such as *Neisseria gonorrhoeae*^36^, *Acinetobacter baumannii*^26^, *N. meningitidis*^44^ and *Pseudomonas aeruginosa*^45^, in recent years. In these studies, LptD was revealed to be one a component of the LPS transport complex, which consists of 7 essential proteins (LptA-LptG), and its physiological function has been suggested to mediate the final translocation of fully synthesized LPS to the cell surface, thus being involved in cell envelope biogenesis^46^. More recently, the crystal structure of LptD has also been determined both in *S. flexneri*^20^ and *S. typhimurium*^21^, and an extraordinarily similar structure that forms a 26-stranded β-barrel has been found. Although these important findings that relate to the structure and function of LptD have been attained, the immunogenic properties and protective immune response against infection of the protein have remained unclear; in particular, there have still been no studies of LptD in *Vibrio* species conducted to date. Therefore, the immunological properties of LptD in *V. parahaemolyticus*, a model *Vibrio* pathogen, were characterized intensively in this study.

Due to the high immunogenicity of LptD observed^19^, we intend to exploit further the potential of LptD for use as a universal vaccine candidate. The initial study was designed to examine whether LptD was associated with host defense because the most attractive feature of OMPs for use as vaccine antigens is their immune properties and exposure on the bacterial surface, which allow OMPs to be accessible to the host immune system^31^,^47^. The positive result of increasing expression of LptD during bacterial exposure to fresh serum was obtained (Fig. 1), suggesting that LptD could play important roles in responses to host defenses, similar to our previous finding that the vaccine candidate OmpW contributed *E. coli* resistance to host defenses^48^,^49^. Although the detailed mechanism of LptD in response to host defenses need be further studied, the results indicated that the OMP is related to immune response, strengthening our interest in further analysis.

Then, we performed bioinformatics analysis based on signal peptide-truncated sequences. LptD was widely distributed among *Vibrio* species. A high level of sequence conservation was also found among *Vibrio* species, particularly with *V. alginolyticus* and *V. harveyi*, two the major *Vibrio* pathogens (Fig. 2a and b). It is apparent that the high conservation and broad distribution of LptD make it possible for them to be targeted as versatile vaccine candidates. Moreover, the sequence identities of *V. parahaemolyticus* with *S. flexneri* and with *S. typhimurium* were as high as 36.9% and 37.6%, respectively. Because the protein structures were more conserved than sequences, when sequence identities are higher than 30% between the query protein and template protein, the quality of the homology model is relatively high^50^. Therefore, based on the structure template of *S. flexneri* LptD, we performed structure modeling using an automated homology prediction server. The three-dimensional structure that formed a 26-stranded β-barrel with several loops and helixes exposed to the external surface was modelled with 100% confidence and 97% sequence coverage (Fig. 3), and it indicated that the modelled structure was very close to the real crystal structure. Based on the modelled structure, antibody epitopes were also predicted, and several linear and discontinued epitopes located on the external surface were obtained (Fig. 2d,e, Supplementary Figs S1 and S2), which were highly conserved among the three major *Vibrio* species (Fig. 2c). These results were consistent with the previous observation that LptD had immunogenic activity^19^. The conserved CTL epitopes among *Vibrio* species were also positively predicted (Supplementary Fig. S3). These data demonstrated that LptD had great potential to be an ideal vaccine antigen.

Thus, to verify this potential, we further determined the protective efficiency by active immune protection assay in a mouse model. As expected, mice that were vaccinated with purified recombinant LptD (Fig. 4a–c) induced strong antibody responses (Figs 4d and 5a) with high specificity (Fig. 4d), and these mice were protected 100% when lethally challenged with *V. parahaemolyticus* (Fig. 5b). This finding suggested that LptD has a very strong ability to stimulate protective immune responses against *V. parahaemolyticus* infection. Previous reports have shown that several characteristics, including surface exposure, conservation among strains and the ability to induce a protective immune response, should be considered to evaluate whether an OMP could be used as a potential vaccine antigen^31^,^51^. Consequently, based on high conservation among *Vibrio* strains, very similar surface epitopes and the strong ability to stimulate protective immune responses, as described above, LptD is clearly a versatile vaccine antigen for protection against *Vibrio* infection. Notably, more efficient elimination of *V. parahaemolyticus* was also observed in the vaccinated mice (Fig. 5c), consistent with previous reports that vaccinated mice had strong bactericidal ability^19^,^52^. In an effective vaccine-based therapy, specific antibodies against OMP could improve complement-mediated clearance of circulating bacteria^52^,^53^, indicating that LptD might also be a potential target for antibody-based immunotherapy against *Vibrio* infections.

Although several OMPs, such as OmpU, OmpK, OmpA(I), OmpA(II), LamB and VP0802 in *V. parahaemolyticus* have previously been shown to be potentially effective vaccine candidates^18^,^19^,^40^,^41^,^42^. LptD is most likely to offer a promising target for developing a vaccine or antibody-based immunotherapy. What led us to hold the opinion was not only the evidence described above but also that LptD plays a particular physiological role, i.e., it is responsible for the final step of LPS transport to the outer leaflet^28^. It is well known that LPS is the major outer surface membrane component of Gram-negative bacteria, which is not only the endotoxin that stimulates host inflammation but is also essential for the vitality of most Gram-negative bacteria^27^,^28^. More importantly, the LPS that surrounds bacteria forms a permeation barrier, which prevents hydrophobic antibiotics from entering the cells, thus conferring antibiotic resistance^25^,^28^,^54^. It has been shown previously that deletion of *lptD* caused a significant decrease in cell LPS^25^,^36^,^45^, and the lack of LPS is commonly accompanied by a decreasing growth rate and increasing sensitivity to antibiotics^26^,^45^,^55^,^56^. The LPS transport proteins have accordingly been considered attractive targets for new drug development^57^,^58^. To evaluate LptD could be a potential target for antibody-dependent therapies, we determined the variations in bacterial growth, LPS level and susceptibility to antibiotics by antibody targeting of LptD rather than knockout of *lptD*, as described previously in several bacteria. Our results showed that antibody targeting of LptD reduced both bacterial growth and LPS content (Fig. 6). More importantly, significantly increased susceptibility to hydrophobic antibiotics (CHL and CIP) with no apparent change in susceptibility to hydrophilic antibiotics (GEN) was observed (Fig. 7) by measuring survival capability. These data were in good agreement with previous findings observed in *lptD*-deletion mutants^26^,^45^,^55^,^56^. To the best of our knowledge, we provided the first evidence that antibodies targeting LptD had similar impacts on bacteria to those observed in *lptD-*deleted strains. Intriguingly, a similar finding was that antibodies targeting TolC, also an OMP, increased susceptibility to CHL in *E. coli* (chloramphenicol, abbreviated CAP in the literature)^39^. The specific antibody-targeting measures not only decreased bacterial proliferation and LPS content but also greatly reduced the dose of used antibiotics, suggesting that combination with immunotherapy and antibiotic treatment might be more efficient than either measure alone. Of course, the therapeutic activity and its possible synergistic effects with antibiotics require further study. Today, multi-drug-resistant Gram-negative bacteria are becoming a serious threat to public health, and unfortunately, the global efforts to develop more efficient antibiotics have not been sufficiently rapid to combat the development of antimicrobial resistance^59^. Alternative strategies, such as the use of antibody-based therapies, would be effective measures and will be widely used to treat bacterial infections in the near future^10^,^11^, and LptD should be a prospective target used in these therapies.

In conclusion, this study showed that LptD, an OMP responsible for final transport of LPS to the outer leaflet, was widely distributed and highly conserved and possessed shared surface epitopes among the several major *Vibrio* pathogens. Furthermore, LptD had very strong ability to introduce response antibodies and provided 100% immune protection against bacterial infection. More importantly, similar to *lptD*-deletion bacteria, antibodies targeting LptD caused dramatically decreased bacterial growth and LPS levels and increased susceptibility to antibiotics. Thus, the protein is a prospective vaccine antigen and therapeutic target for the prevention and control of *Vibrio* infection.

## Materials and Methods

### Bacterial strains and growth conditions

Bacterial strains of *V. parahaemolyticus* serotype O3:K3 (strain RIMD 2210633), *E. coli* BL21 and *E. coli* DH5α, and plasmid pET-28a(+) are maintained in our laboratory. *V. parahaemolyticus* cells were cultured in marine LB (MLB) medium (LB medium containing 3% NaCl) at 37 °C. *E. coli* were cultured at 37 °C in LB broth or agar medium added with 50 μg/mL kanamycin if needed.

### Bacteria treatment and real-time quantitative PCR

*V. parahaemolyticus* cells were treated by incubation with 15% (v/v) human sera for 1 h at 37 °C. After incubation, the intact cells were collected by centrifugation at 6000 g for 10 min. Real-time quantitative PCR analysis of the expression of *lptD* was performed as described in our previous work^14^. Primer pairs of F (CCCGAACATTCCAGACGAGACAA) and R (CGTACTGAACGCCACCGTGATAGA) were synthesized and applied for the amplification of *lptD*. The simultaneous amplification of the 16 S rRNA gene of *V. parahaemolyticus* with primer pairs of F (GCCTTCGGGAACTCTGAGACAG) and R (GCTCGTTGCGGGACTTAACCCAA) was used as an internal control. Real-time quantitative PCR was conducted using a real-time PCR amplification system (Bio-Rad, USA) and SYBR Premix Ex Taq™ kit (Takara, China).

### Bioinformatics analysis

A homology search of LptD was performed with BLAST in UniProt (http://www.uniprot.org/blast). ClustalW was used to analyze multiple alignment of protein sequences (https://npsa-prabi.ibcp.fr/cgi-bin/align_clustalw.pl), and a phylogenetic tree was constructed using Maximum Likelihood in MEGA6.0. Three-dimensional models were generated using automated homology prediction through the Phyre2 online server^60^ (http://www.sbg.bio.ic.ac.uk/~phyre2). Antibody epitopes predicted by ElliPro server^61^ (http://tools.immuneepitope.org/ellipro) and DiscoTope2.0^62^ (http://tools.immuneepitope.org/stools/discotope/discotope.do), both based on the modelled three dimensional structure of LptD. Cytotoxic T lymphocyte (CTL) epitope prediction was performed by the online software CTLPred based on the SVM method^63^ (http://www.imtech.res.in/raghava/ctlpred).

### Cloning, expression and purification of LptD

Cloning, expression and purification of LptD were conducted as described previously^19^. In brief, the primer pair of F (CGCCATATGATGCAACATTTCTCC) and R (CCGCTCGAGTTAATTGTTCAAATAG) (underlined sequences are *Nde*I and *Xho*I restriction enzyme sites, respectively) was designed to amplify *lptD* gene by PCR. Then, the PCR product was cloned into plasmid pET28a(+) and the recombinant plasmids were transformed into *E. coli* DH5α. The positive recombinant transformants, which selected by LB agar plates containing 50 μg/mL of Kanamycin and verified by sequencing, were cloned into *E. coli* BL21 (DE3). The *E. coli* BL21 (DE3) cells harbored recombinant plasmid were cultured with antibiotics-containing medium and induced upon addition to Isopropyl β-D-1-Thiogalactopyranoside (IPTG) at a final concentration of 0.5 mM. Cells were harvested and lysed by sonication. The insoluble inclusion bodies of the recombinant protein were separated by centrifugation at 12,000 g for 10 min at 4 °C. Then inclusion bodies were washed twice with lysis buffer containing 4 M urea and 0.5% Triton X-100. After centrifugation at 12,000 g for 10 min, the pellets were collected and added to loading buffer. The resulting samples were submitted to SDS-PAGE, and the major protein bands corresponding to recombinant LptD were excised. Gel bands were cut into small pieces, then added the PBS for grinding. The result proteins were leached from the suspension overnight. Protein expression and purification were verified by SDS-PAGE, which carried out in 12% slab SDS-gels in a standard procedure and protein bands were displayed by staining with Coomassie Brilliant Blue R-250. Protein concentration was determined using BCA method.

### Dot-ELISA assay of antiserum titer

An aliquot of 1 μg of purified recombinant LptD was loaded onto PVDF membrane, and was then blocked overnight with 5% bovine serum albumin (BSA) at 4 °C. After rinsing the membrane, the proteins were probed with mouse antiserum collected from immunized and control mice as primary antibodies, and an HRP-rabbit anti-mouse antibody was used as the secondary antibody. The conjugate was displayed using an enhanced chemiluminscent imaging system (ChampChemi^TM^, China).

### Extraction of membrane proteins and Western blotting analysis

*V. parahaemolyticus* were cultured and harvested during grown to an OD600 of 0.8 by centrifugation. The pellets were then resuspended in lysis buffer (50 mM Tris-HCl, 150 mM NaCl, pH 7.4) and broken by ultrasonication on ice. After that, removed cell debris by centrifugation at 12,000 g for 15 min, and the supernatants were collected and ultracentrifuged at 100,000 g for 1 h at 4 °C. The resulting pellets that correspond to membrane protein samples were resuspended in lysis buffer containing 1% Triton X-100 and stored at −20 °C until use. For Western blotting, the membrane proteins were firstly electrophoresed in 12% slab SDS-gels, and then were transferred to PVDF membrane. Next, blocked membrane overnight with 5% skim milk in TBST (25 mM Tris, 150 mM NaCl, and 0.05% (v/v) Tween-20, pH 7.4) at 4 °C. Then, the membrane were washed three times with TBST and incubated with mouse antibody against LptD for 2 h with gentle shaking. After rinsing three times, the membrane incubated with anti-mouse secondary antibody for 2 h also with gentle shaking. The membranes were then rinsed, and the band was displayed by using a chemiluminscent imaging system (ChampChemi^TM^, China).

### Vaccination of mice and active immune protection assay

ICR mice were purchased from Center for Animal Research at Zhejiang Chinese Medical University, Hangzhou, China. The vaccination of mice and active immune protection assay were performed as described in our previous work^48^. Briefly, 50 μg of recombinant LptD emulsified with incomplete Freund’s adjuvant were immunized into each ICR mouse (6–8 weeks old). Protein in a mass of 40–50 μg was used 3 times for booster immunizations at intervals of 7 days. Another 6 ICR mice were immunized with PBS buffer emulsified with incomplete Freund’s adjuvant used as a control. On the seventh day of the final immunization, 50 μL of blood were collected from each mouse and titers of antibodies against LptD in serum were determined by dot enzyme-linked immunosorbent assay (dot-ELISA). One week later, all of the mice in the two groups (immunization and control) were challenged intraperitoneally (i.p.) with 2.5 × 10^7^ CFU of *V. parahaemolyticus*. After challenge, the deaths of the mice were observed for 10 days and mortality was recorded daily. Ten days later, antiserum from each immunized mouse was collected and stored at −80 °C until use.

### *In vivo* assay of the clearance of bacteria

The *in vivo* assay of the clearance of bacteria in mice was conducted by tail vein injection of 10^6^ CFU of *V. parahaemolyticus* in two groups of mice (3 mice in each group), as described previously^14^. Four hours post-injection, 100 μL of blood were collected from mouse tail veins and added to 100 μL sterile of PBS containing heparin. The blood samples were diluted and spread on LB agar plates to enumerate the surviving bacteria.

### Measurement of bacterial growth

Antiserum against LptD from immunized mice and normal serum from mice without immunization were used to determine their impact on bacterial growth. To avoid serum complement mediated-killing, both sera were inactivated by incubating the sera in a 56 °C water bath for 30 min prior to use. A single colony of *V. parahaemolyticus* was chosen and inoculated into 5 mL of MLB medium containing 50 μL of heat-inactivated normal serum and antiserum, respectively, grown at 37 °C with shaking at 180 rpm. Two cultures were separately collected at intervals, and optical density at 600 nm was determined by spectrophotometry (Beckman DU-800, USA).

### Extraction and visualization of LPS

A hot aqueous-phenol method was applied for the extraction of LPS from *V. parahaemolyticus* cells, as described previously^64^ with slight modification. Bacterial cultures were prepared in 5 mL of MLB medium supplemented with 50 μL of normal serum and antiserum (the antibody titer against LptD was 1:10000), respectively. Bacterial cells were harvested and washed twice with 0.85% NaCl and then diluted to 2 × 10^8^ CFU/mL with 0.85% NaCl. The cells were lysed by repeated freezing-thawing at −80 °C and 37 °C at least 3 times. Then, an equal volume was added of 90% hot aqueous-phenol and incubated for 30 min at 65 °C with occasional shaking. After incubating, the samples were cooled in cold water and maintained at 4 °C overnight. The samples were centrifuged at 10000 g for 10 min at 4 °C. The supernatants were collected and dialyzed in 0.85% NaCl to remove the phenol. Subsequently, the samples were collected and concentrated by PEG 6000. Five to twenty microliters of samples were used for electrophoresis on 12% polyacrylamide gel. LPS was visualized by silver staining, using a standard protocol.

### Antibiotic susceptibility analysis of *V. parahaemolyticus* by targeting the antibody

Three types of antibiotics – gentamicin Sulfate (GEN), chloramphenicol (CHL) and ciprofloxacin (CIP) (products of Solarbio) – were used for this assay. The minimum inhibitory concentrations (MICs) of these antibiotics were first determined by broth microdilution methods in a procedure described by CTSI^65^. In brief, bacteria were cultured in MLB medium to an OD_600_ of 0.5, and then the cultures were diluted with fresh medium to an OD600 of 0.3. One hundred microliters of bacteria were separately added to every well that contained 100 μL of MLB medium supplemented with different concentrations of antibiotics. Then, the 96-well plate was placed in an incubator at 37 °C for 24 h. After determination of the MIC of each antibiotic, antibiotic susceptibility analysis of *V. parahaemolyticus* targeting by LptD antibody was performed as described previously^39^ with minor modifications. Briefly, 5 mL of bacterial cells (grown to an OD_600_ of 0.5) were harvested by centrifugation, and the pellets were resuspended in 200 μL of PBS and subsequently incubated with 100 μL of normal serum and antiserum (antibody titer against LptD was 1:10000), respectively, for 1 h at 37 °C. Bacterial cells were then recollected by centrifugation and adjusted to an OD_600_ of 0.3 with MLB medium. One hundred microliters of bacterial suspensions were added to every well of 96-well plates containing the same volume of MLB medium supplemented with 5 μL of normal serum/antiserum and with 1/8, 1/4, or 0 MIC of antibiotics, respectively. The plates were maintained in an incubator at 37 °C, and the OD_600_ values of bacterial suspensions were determined at intervals with a Multimode Microplate Reader (Thermo Scientific Varioskan™ Flash, USA).

### Statistical analysis

Student’s *t*-test was used for the evaluation of the alternation in LptD expression, bactericide in mice and bacterial growth. Data are expressed as the mean ± SD (n = 3). The statistical significance was set at a *P* value less than 0.01 (*P* < 0.01).

### Ethics statement

ICR mice used in the study were purchased from Center for Animal Research at Zhejiang Chinese Medical University, Hangzhou, China. All animal experimental protocols of the study are in accordance with the guidelines from the China Council on Animal Care and approved by the guidelines of the Ethics Committee of Animal Experiments at Zhejiang Sci-Tech University. All mice were sacrificed under ether anesthesia and all efforts were made to minimize suffering.

## Additional Information

**How to cite this article**: Zha, Z. *et al*. LptD is a promising vaccine antigen and potential immunotherapeutic target for protection against *Vibrio* species infection. *Sci. Rep.* **6**, 38577; doi: 10.1038/srep38577 (2016).

**Publisher's note:** Springer Nature remains neutral with regard to jurisdictional claims in published maps and institutional affiliations.

## Supplementary Material

Supplementary Information
