# Supplementary material for: LptD is a promising vaccine antigen and potential immunotherapeutic target for protection against Vibrio species infection
Source: Sci Rep. 2016 Dec 6;6:38577. doi: 10.1038/srep38577 (PMC5138612; doi:10.1038/srep38577)
Supplement: Supplementary Information [file srep38577-s1.pdf]

Supporting information

LptD is a promising vaccine antigen and immunotherapeutic target for protection against Vibrio species infection

Zhenzhong Zha[1,2#], Chuchu Li[1,2#], Weiyan Li[1,2], Zhicang Ye[1,2] and Jianyi Pan[1,2*]

[1] School of Life Sciences, Zhejiang Sci-Tech University, Hangzhou 310018, China

[2] Zhejiang Provincial Key Laboratory of Silkworm Bioreactor and Biomedicine, Hangzhou 310018, China

***Corresponding author:**

Dr. Jianyi Pan, School of Life Sciences, Zhejiang Sci-Tech University, Hangzhou 310018, China; Zhejiang Provincial Key Laboratory of Silkworm Bioreactor and Biomedicine, Hangzhou 310018, China., Tel: +86 571 86843747, Fax: +86 571 8684 3745, E-mail: jianyi.pan@zstu.edu.cn

[#] These authors contribute equally to this work.

Legends of Supplementary Figures

Fig. S1 Three dimensional structures showed linear antibody epitopes of (**a**) YLNSDKKYQDDS (286-297)(score of 0.77), (**b**) IPDETTNYS (585-593)(score of 0.648), and (**c**) LENLDT (656-661)(score of 0.507); epitope residues were in yellow. Epitopes were predicted by ElliPro based on the modelled three dimensional structure of LptD.

Fig. S2 Chart review of discontinuous B cell epitopes predicted by DiscoTope (http://tools.immuneepitope.org/stools/discotope/discotope.do) which was based on predicted three dimensional structure of LptD. Prediction threshold was set at -1.

Fig. S3 Homology alignment of top 5 score of CTL epitopes of V. parahaemolyticus LptD among different Vibrio species. The CTL epitopes were predicted by CTLPred server based on SVM methods. Identical residues were in red and strongly similar residues were in green. The epitopes were listed with score from high to low. Peptides boxed with dotted line were distributed inside of cell outer membrane.

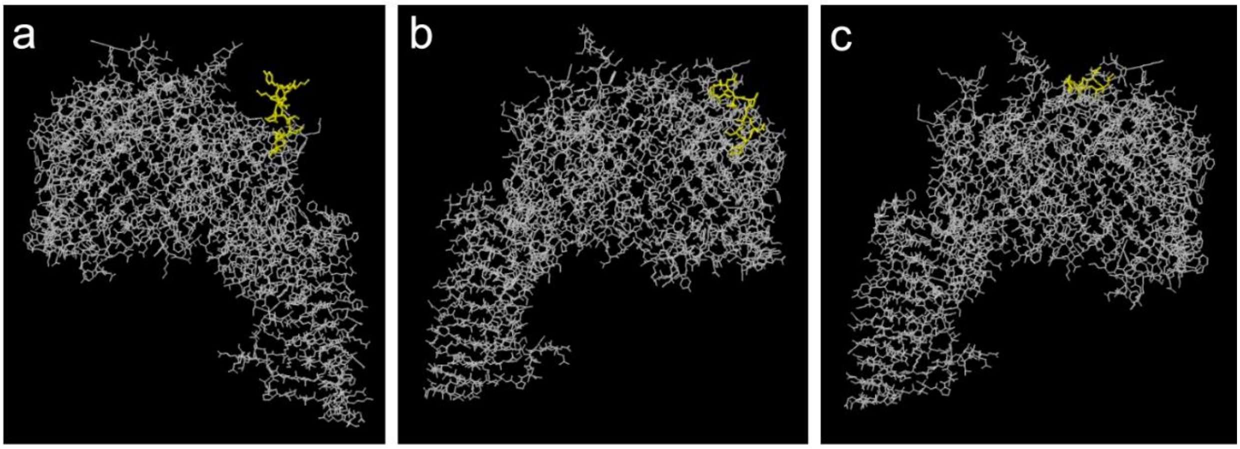

Fig. S1

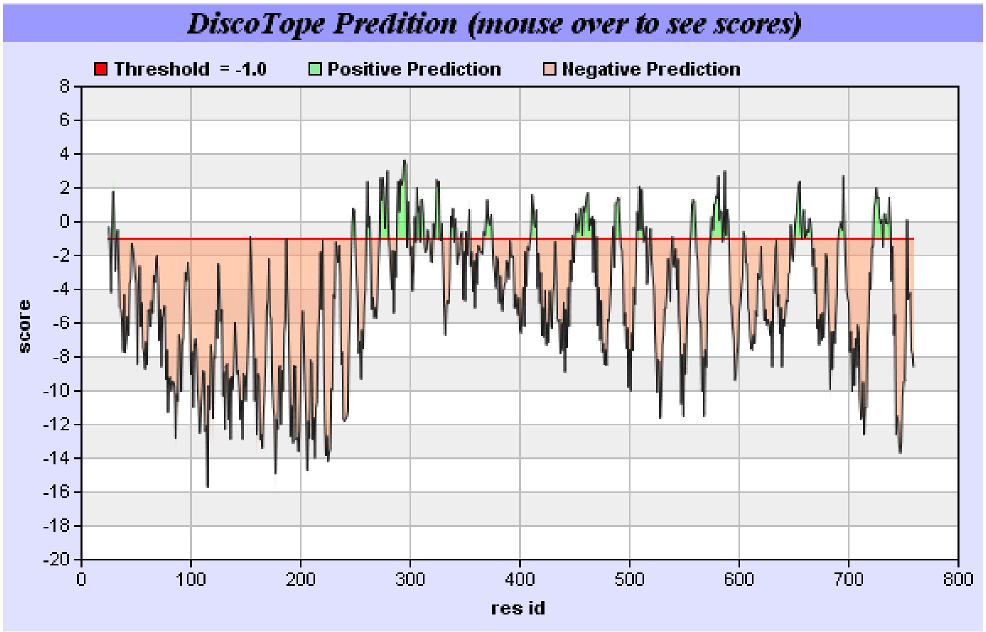
DiscoTope Predition (mouse over to see scores)
Threshold = -1.0
Positive Prediction
Negative Prediction
score
8
6
4
2
0
-2
-4
-6
-8
-10
-12
-14
-16
-18
-20
0
100
200
300
400
500
600
700
800
res id

Fig. S2

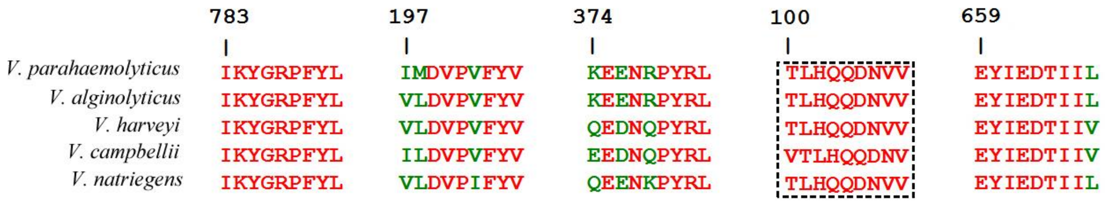
783
197
374
100
659
V. parahaemolyticus IKYGRPFYL IMDVPVFYV KEENRPYRL TLHQQDNVV EYIEDTIIL
V. alginolyticus IKYGRPFYL VLDVPVFYV KEENRPYRL TLHQQDNVV EYIEDTIIL
V. harveyi IKYGRPFYL VLDVPVFYV QEDNQPYRL TLHQQDNVV EYIEDTIIV
V. campbellii IKYGRPFYL ILDVPVFYV EEDNQPYRL VTLHQQDNV EYIEDTIIV
V. natriegens IKYGRPFYL VLDVPIFYV QEENKPYRL TLHQQDNVV EYIEDTIIL

Fig. S3
